# Myocarditis and pericarditis recovery following smallpox vaccine 2002–2016: A comparative observational cohort study in the military health system

**DOI:** 10.1371/journal.pone.0283988

**Published:** 2023-05-08

**Authors:** Renata J. M. Engler, Jay R. Montgomery, Christina E. Spooner, Michael R. Nelson, Limone C. Collins, Margaret A. Ryan, Clara S. Chu, John E. Atwood, Edward A. Hulten, Ahlea A. Rutt, Dacia O. Parish, Bruce M. McClenathan, David E. Hrncir, Laurie Duran, Catherine Skerrett, Laurie A. Housel, Janet A. Brunader, Stephanie L. Ryder, Connie L. Lohsl, Brian A. Hemann, Leslie T. Cooper

**Affiliations:** 1 Immunization Healthcare Division, Defense Health Agency, Falls Church, Virginia, United States of America; 2 Walter Reed National Military Medical Center, Bethesda, Maryland, United States of America; 3 Uniformed Services University of the Health Sciences, Bethesda, Maryland, United States of America; 4 MDC Global Solutions, LLC, Manassas, Virginia, United States of America; 5 University of Virginia, Charlottesville, Virginia, United States of America; 6 Naval Medical Center, San Diego, California, United States of America; 7 Cardiology Service, Walter Reed National Military Medical Center, Bethesda, Maryland, United States of America; 8 Womack Army Medical Center, Fort Bragg, North Carolina, United States of America; 9 Wilford Hall Ambulatory Surgical Center, Lackland Air Force Base, San Antonio, Texas, United States of America; 10 Cardiocare, LLC, Chevy Chase, Maryland, United States of America; 11 Department of Cardiovascular Medicine, Mayo Clinic, Jacksonville, Florida, United States of America; University of Dundee, UNITED KINGDOM

## Abstract

**Objectives:**

(1) Characterize the initial clinical characteristics and long-term outcomes of smallpox vaccine-associated hypersensitivity myocarditis and pericarditis (MP) in United States service members. (2) Describe the process of case identification and adjudication using the 2003 CDC nationally defined myocarditis/pericarditis epidemiologic case definitions to include consideration of case-specific diversity and evolving evidence.

**Background:**

Between 2002 and 2016, 2.546 million service members received a smallpox Vaccinia vaccine. Acute MP is associated with vaccinia, but the long-term outcomes have not been studied.

**Methods:**

Records of vaccinia-associated MP reported to the Vaccine Adverse Event Reporting System by vaccination date were adjudicated using the 2003 MP epidemiologic case definitions for inclusion in a retrospective observational cohort study. Descriptive statistics of clinical characteristics, presentation, cardiac complications, and time course of clinical and cardiac recovery were calculated with comparisons by gender, diagnosis and time to recovery.

**Results:**

Out of over 5000 adverse event reports, 348 MP cases who survived the acute illness, including 276 myocarditis (99.6% probable/confirmed) and 72 pericarditis (29.2% probable/confirmed), were adjudicated for inclusion in the long-term follow-up. Demographics included a median age of 24 years (IQR 21,30) and male predominance (96%). Compared to background military population, the myocarditis and pericarditis cohort had a higher percentage of white males by 8.2% (95% CI: 5.6, 10.0) and age <40 years by 4.2% (95% CI: 1.7,5.8). Long-term follow-up documented full recovery in 267/306 (87.3%) with 74.9% recovered in less than a year (median ~3 months). Among patients with myocarditis, the percentage who had a delayed time to recovery at time of last follow-up was 12.8% (95% CI: 2.1,24.7) higher in those with an acute left ventricular ejection fraction (EF) of ≤50% and 13.5% (95% CI: 2.4,25.7) higher in those with hypokinesis. Patient complications included 6 ventricular arrhythmias (2 received implanted defibrillators) and 14 with atrial arrhythmias (2 received radiofrequency ablation). Three of 6 patients (50%) diagnosed with cardiomyopathy had clinical recovery at their last follow-up date.

**Conclusions:**

Hypersensitivity myocarditis/pericarditis following the smallpox vaccine is associated with full clinical and functional ventricular recovery in over 87% of cases (74.9% <1 year). A minority of MP cases experienced prolonged or incomplete recovery beyond 1 year.

## Introduction

Smallpox or variola, a virulent double-stranded DNA orthopoxvirus, is designated as a Category A bioterrorism infectious agent due to the high infection-associated morbidity and mortality as well as the risk for rapid aerosol transmission [1–4]. Accordingly, in December 2002, the Department of Defense (DOD) Smallpox Vaccine (SPV) Immunization Program [4], under a mandatory force health protection policy, initiated the delivery of vaccine doses to potentially at-risk service members.

Myocarditis and pericarditis (MP) following live, attenuated, and replicating vaccinia vaccination has been one of the more prominent adverse events described within the DOD immunization program [5–7]. A Military Health System (MHS) vaccine safety surveillance and care support registry program was established in 2002–2003 under a collaboration between the Centers for Disease Control and Prevention (CDC) Vaccine Adverse Event Reporting System (VAERS) and a Congressionally sponsored program known as the Vaccine Healthcare Centers Network (VHCN) [8]. The purpose of this program was to enhance military VAERS evaluation, reporting, and outcomes. This program provides enhanced follow-up enabling better data quality for subsequent adjudication. In addition, the rapid deployment of the smallpox vaccine presented a challenge for optimizing the quality of clinical screening, medical exemption management, and early recognition of adverse events even if not previously identified. The majority of the medical community had no prior experience with SPV and potential adverse events. An augmented case assessment and care process was established within the VHCN’s multi-site regional platform (linked to MHS facilities), enabling more precise myocarditis/pericarditis diagnostic adjudication (probable or confirmed rather than suspected) [9]. Cases that met a level of certainty consistent with 2003 epidemiologic consensus case definitions were entered into an MHS clinical MP registry [10].

Using background incidence data from the DOD Immunization Registry and Defense Medical Surveillance System [11], MP relative risk association with SPV beyond background rate was established early in the program [5–7]. Clinical evaluation and specific diagnostic testing (blood, cardiac tissue when available) was consistent with an immune inflammatory mechanism suggesting a hypersensitivity (eosinophilic) inflammatory mechanism rather than direct viral infection of myocardium [12–14].

The current study data draws from the collective MP case management and review experience of the Immunization Healthcare Division, Defense Health Agency. VAERS cases identified reflect the vaccine safety surveillance efforts of a program that immunized over 2.5 million service members who met the occupational/readiness eligibility criteria and were without clinical contraindications to live attenuated vaccinia containing vaccines [15, 16].

This paper will focus on the following objectives:

(1) Describe the clinical characteristics and long-term outcomes of smallpox vaccine-associated myocarditis and pericarditis (MP) cohort of United States (US) Department of Defense (DOD) service members.
(2) Describe the process of case identification and adjudication using the 2003 CDC nationally defined myocarditis/pericarditis epidemiologic case definitions to include consideration of case specific diversity and evolving evidence.

## Methodology

This is a retrospective comparative observational cohort study of individuals who, after receiving a dose of vaccinia vaccine between December 1, 2002 through December 31, 2016 (calf-lymph derived Dryvax^®^ used December 2002 through February 2008, followed by the cell-culture derived ACAM2000^®^), developed a clinical presentation that met criteria for vaccine-associated myocarditis or pericarditis (Table 1) [10]. Based on a 2015 prospective study of MP after smallpox vaccine, the cases associated with each of the vaccines are considered equivalent given that both contained the same live attenuated virus and the clinical presentations were comparable [17]. The Institutional Review Board and Human Use Committee under the Department of Research Programs (Walter Reed National Military Medical Center, Bethesda, MD) approved the research study described in this manuscript (Protocol Number: 20525). The approval included medical record data extraction into anonymized data sets that were analyzed for publication. The content of the manuscript was reviewed for clearance prior to submission for publication by the Department of Research Programs.

**Table 1:** Myocarditis/pericarditis epidemiologic case definitions: Centers for Disease Control and Prevention (CDC) [10, 18].

Myocarditis	Pericarditis
**SUSPECTED MYOCARDITIS**: Presence of symptoms (dyspnea, palpitations, chest pain) and either an abnormal ECG OR any evidence of focal or diffuse depressed left ventricular (LV) function of uncertain age by an imaging study.	**SUSPECTED PERICARDITIS**: Typical chest pain (made worse by supine position, improvement with leaning forward, pleuritic, constant) AND no evidence of alternate cause of such pain.
**PROBABLE MYOCARDITIS**: Symptoms plus elevated cardiac enzymes OR new onset depressed LV function by imaging OR abnormal imaging consistent with myocarditis (MRI with gadolinium, gallium 67 scanning, anti-myosin antibody scanning)	**PROBABLE PERICARDITIS**: SUSPECTED PERICARDITIS criteria plus one or more of the following: Pericardial friction rub on auscultation OR ECG abnormality OR echocardiogram revealing an abnormal pericardial effusion.
**CONFIRMED MYOCARDITIS**: Histopathological evidence of myocarditis by endomyocardial biopsy or autopsy.	**CONFIRMED PERICARDITIS**: Histopathological evidence of pericardial inflammation in pericardial tissue from surgery or autopsy.

In order to provide a background context for the Military Health System MP VAERS cases, a search of the VAERS public health database (December 1, 2002- December 31, 2016) using the CDC Wonder search tool was conducted focusing on all SPV associated VAERS followed by stepwise limiting criteria [19]. Possible cardiac adverse event reports were identified using the MedDRA (Medical Dictionary for Regulatory Activities) terms outlined in S1 Table [20].

The adjudication process for adverse events following immunization being classified as myocarditis or pericarditis was performed as part of the MHS public health efforts for enhanced military VAERS surveillance for MP cases using the nationally accepted epidemiologic case definition [9, 18]. The process of adjudication did not consider 2 diagnoses separately but rather classified cases by what was considered the more severe/impacting diagnosis (myocarditis) even if features of pericarditis were present.

Data sources included VAERS and MHS records (with access to Veterans Administration medical records) and the Public Health Surveillance activities of the Immunization Healthcare Division (IHD), when available. Data included case-specific demographics, clinical history, examinations, laboratory and imaging results, diagnoses, and follow-up outcomes. With variable availability and content of case-specific information, raw data was extracted and redacted for review and categorization into structured variables for analysis. Composite clinical data definitions were constructed for analysis from raw data where needed.

VAERS reporting from the MHS was facilitated and enhanced (in terms of data content) through a centralized immunization healthcare call center with 24/7 access to clinical immunization safety and efficacy support teams providing both direct and indirect (virtual) outreach. The network of regional support sites with a centralized informatics platform linked to the VAERS database provided assistance with clinical care coordination as well as assistance with documenting adverse events including VAERS submissions. It is noteworthy that this process resulted in greater precision of MP diagnosis compared to CDC civilian MP VAERS data [9, 16].

S2 Table includes the specific definitions reflecting aggregated clinical documentation to describe symptoms clustered as cardiac, systemic, gastrointestinal, upper respiratory, demographics, and case-specific variables. These variables include other vaccines given, clusters of medication groups for acute treatment (non-steroidal anti-inflammatory (NSAID), myocardial infarction (MI) treatment, gastrointestinal treatment), cardiac risk factors, and dyslipidemia. Body mass index (BMI) was not included in the composite cardiac risk factors variable since there existed the potential for error in categorization given the data that young, physically active service members often have an elevated calculated BMI but normal or low measured body fat [21]. Recent or current tobacco use is detailed as a separate variable.

Because the Military Health System represents a national and global platform of care delivery (including civilian and foreign national resources), there was considerable variability in documentation of laboratory testing. Information about specific assays (with definition of normal ranges) as well as quantitative values for markers for systemic inflammation (erythrocyte sedimentation rate/ESR and/or C-reactive protein/CRP) and cardiac specific injury (e.g., troponin, creatine kinase-myoglobin binding/CKMB cardiac isoenzyme) was not consistently present in all files reviewed. Composite variables were defined as follows:

- **Systemic Inflammation Markers**: ESR and/or CRP elevated or abnormal qualitative data or quantitative data with normal ranges for laboratory provided.
- **Objective measures of cardiac injury**: Qualitative or quantitative documentation of an abnormal or elevated troponin, troponin I, troponin T, and/or CKMB.

Variables reflecting findings from reported electrocardiogram (ECG) and imaging study results (echocardiogram, angiography, cardiac magnetic resonance (CMR), indium 111- tagged white blood cell scan) are detailed below:

- **ECG**: Classified as abnormal or normal (including early repolarization that did not evolve to an abnormal reading).
- **Ejection fraction** (EF): Results available ranged from qualitative to quantitative with normal poorly defined as 50–55%. The population consisted of young, healthy, physically active service members. Military cardiologist recommendations considered an EF (when available) of 51–55% as low normal; therefore, less than or equal to 50% was considered abnormal. The majority of the data available referenced the left ventricular EF or did not specify.
- **Hypokinesis**: Abnormal wall motion in any location of the heart (with variable data available in each case) was classified as hypokinesis yes or no. Available records often did not specify if the hypokinesis was regional or global. Military cardiologists noted that if the EF is 50% or less with symptoms, the annotation of hypokinesis may not be recorded because hypokinesis is implied in the low EF and is usually global.
- **Non-obstructive coronary artery disease**: Coronary artery imaging results described as having some evidence of coronary artery disease but described as not relevant to the acute clinical presentation.
- **CMR Abnormal**: The majority of cases did not have detailed data from a CMR or indium scan; results represent a physician/cardiology note referencing that it was normal or abnormal (consistent with findings associated with myocarditis and/or pericarditis).

Special testing data based on information available in the VAERS documentation and/or other records relevant to the cases described included polymerase chain reaction (**PCR**) assessment of possible infectious etiology from blood and/or tissues.

Data regarding four death cases were summarized from existing records containing case review data and a multidisciplinary causality assessment performed as part of the ongoing public health surveillance process supported by the legacy Vaccine Healthcare Centers Network and Military Vaccine Agency (current Immunization Healthcare Division, Defense Health Agency) between 2003 and 2012.

### Follow-up data standardization

Estimates for time to recovery and time to last documented follow-up demonstrating recovery or ongoing clinical issues considered impacting by the patient and/or provider were obtained from redacted sequential medical records and/or documentation by the public health adverse events tracking process internal to the IHD. Dates represent estimates drawn from clinical notes related to medical encounters and/or telephonic communication documenting recovery or ongoing impacting symptoms and/or related medical issues. Definitions used for standardizing the data related to outcomes are detailed below:

- **RECOVERED**: Clinical recovery (no or minimal symptoms with no impact on the ability to work, quality of life and/or overall functional capacity) with documentation of normal cardiac function and lacking evidence of ongoing cardiac injury. If there was no specific documentation of follow-up cardiac functional capacity recovered was defined as several years without symptoms, normal annual physical examinations, ability to perform militarily operational duties and passing physical fitness tests.
- **NOT RECOVERED/PROLONGED IMPACT**: More than 1 year of ongoing and/or recurrent clinical symptoms with impact on activity and functional capacity and/or long-term complications requiring repeated medical evaluation and/or treatment. The “No Recovery” date reflects the last follow-up documentation (usually greater than 5 years from the vaccination date) with clinical issues and concerns reflecting that the individual did not consider themselves recovered to baseline.
- **INSUFFICIENT FOLLOW-UP**: Insufficient data available within 8 weeks following SPV vaccination to characterize clinical symptoms and cardiac function needed to categorize status and/or no follow-up data beyond 3 months to support the return to normal functional capacity. Individuals were often reservists (only receiving some care in the MHS) or were separated from service (trainees) so no civilian follow-up was available.

Data for cohort comparison to the relevant background immunized population (service members with SPV vaccination dates between 2002–2016) was prepared by the Armed Forces Health Surveillance Division (Public Health Directorate, Defense Health Agency) using the Defense Medical Surveillance System (DMSS) [11]. The DMSS data set, first established in 1993, does not provide separate categories for race and ethnicity so the Hispanic category could include White or Black with Caucasian or African American potentially including Hispanic. Other races were included in the category “Other.”

### Statistical method

Frequencies, percentages, and comparisons of percentage differences, along with their respective 95% confidence intervals (CI), are provided. The Miettinen-Nurminen method of calculating confidence intervals was implemented because it has coverage probabilities close to nominal levels for moderate to large samples (at least 30 in each sample) [22, 23]. Rates of MP cases in different vaccine cohorts per 100,000 vaccinees (with 95% CI) and relative rates were computed based on the Poisson distribution. Comparisons between myocarditis and pericarditis and by gender at baseline were evaluated using chi-square or Fisher’s exact tests for demographics, clinical symptoms, physical findings, and laboratory testing results. Measures of cardiac injury acutely were compared between short recovery (<1 year) and prolonged (≥ 1 year) or no documented full recovery (after 1 year) among those who had follow-up data available.

Descriptive non-parametric statistics, including the median, interquartile range (IQR), and range are provided for continuous variables, including age at vaccination, time (in days) to onset (cardiac symptoms, diagnosis) at baseline and time to full recovery and time to follow-up in those who had follow-up data available. Statistical inference for these variables was performed using the Wilcoxon Rank Sum test. Significance was declared at the 0.05 level. All statistical analyses were performed using SAS^®^ software version 9.4 of the SAS^®^ system for Windows. Copyright © [2002–2012] by SAS Institute Inc [24].

## Results

**Fig 1:**
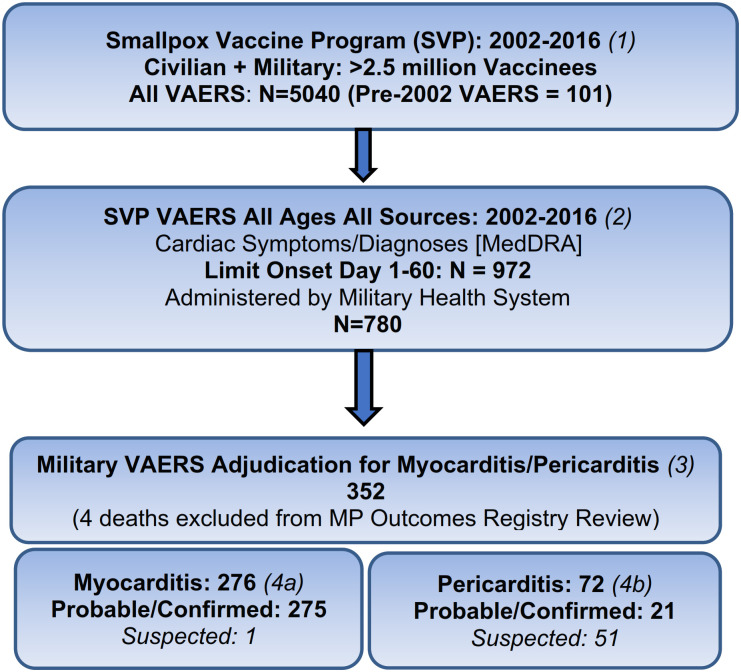
Smallpox vaccine program 2002–2016 date of immunization: Vaccine Adverse Event Reporting System (VAERS) data available with iterative database searches ldentifying subsets of cases with new onset cardiac symptoms and then meeting the 2003 epidemiologic case definition for myocarditis/pericarditis. Box 1: Vaccination Dates: Dec 2002–2016: Data Source: CDC/FDA VAERS Database using CDC Wonder Search tool. Accessed June 2020. https://wonder.cdc.gov. Box 2: Cardiac Symptoms/Diagnoses by MedDRA Terms (see S1 Table): All Ages, Locations, No Limits (1081) = then Limit Symptom Onset Day 1–60 = then Limit to Military. Box 3: Final Cohort of myocarditis and pericarditis with smallpox vaccine association using CDC case definitions (2003: See Table 1). Box 4a and 4b (left to right): Myocarditis and Pericarditis cases detailed by level of certainty for diagnostic accuracy based on criteria in CDC case definitions with no other etiology identified.

From the cohort of service members with smallpox vaccine associated VAERS and vaccination dates between December 2002 through 2016, there were over 5000 reports. There were 1081 VAERS cases with 1 or more new-onset cardiac symptoms. As illustrated in Fig 1, the final cohort of post-smallpox vaccine myocarditis and pericarditis cases (in the MHS) adjudicated as meeting the published epidemiologic case definitions for MP (Table 1) included 348 cases that survived the acute illness and 4 that died in temporal association with their acute illness. Of surviving myocarditis patients, 99.6% (275/276) were categorized as probable or confirmed compared to 29.2% (21/72) of pericarditis patients. The remaining MP patients (52/348) were categorized as suspected. Seventy-five percent of the multidisciplinary adjudicated MP cases (Table 2) described were generated through stimulated direct contact by the DOD program.

**Table 2:** Distribution of myocarditis and pericarditis cases by source of initial contact (including 4 deaths).

#	Sources for Initial Case Identification	N = 352 (%)	Description of Process Supporting Adverse Events Care & Reporting
1	Primary Care Provider or Specialist	175 (49.7)	DOD or civilian direct contact of IHD clinical staff via electronic mail or direct telephone outreach
2	DOD Vaccine Clinical Support Consultation 24/7	50 (14.2)	Call Center 877-GETVACC (438–8222) providing 24/7 global access for vaccine clinical support including assistance with filing VAERS
3	Patient/Family Member Self-Referral	25 (7.1)	Direct to staff of Immunization Healthcare Division (IHD) Clinical Site via walk-in, phone, or email
4	Other Federal Agencies	13 (3.7)	Direct contact of Immunization Healthcare Division staff, includes Armed Forces Health Surveillance Center
**5**	**Percent Non-VAERS Sources**	(74.7)	Cases submitted to VAERS by MHS platform
6	VAERS Source, Primary review	89 (25.3)	Systematic iterative review of VAERS (identified VAERS cases not accrued through acquisition methods 1–4 above)

The demographics of the surviving MP cohort (Table 3) includes median age of 24 years (IQR 21,30), predominance of males (96%) and white race (82.5%). In comparison to the background immunized population (2.546 million), the pattern of age distribution associated with MP following SPV was 96.2% falling within the younger population (<40 years) versus 92.0% in the background population giving a difference of 4.2%; [95% CI: 1.7,5.8). White males were significantly increased in the myocarditis and pericarditis cohort by 8.2% (95% CI: 5.6,10.0) and compared to other races by 18.5% (95% CI: 14.1,22.1).

**Table 3:** Myocarditis/Pericarditis (MP) cohort (alive 8 weeks post SPV with data for adjudication) demographics compared to the total immunized population by the Military Health System (MHS): December 1, 2002-December 31, 2016.

Comparison of MP Cohort with Immunized Population	MP Cases N (%)	MHS Total N X 10^3^ (%)	Comparison
**Total Cases**	**348**	**2546**	
**SMALLPOX Vaccine (SPV)**			
**Dryvax^®^ MP**	169 (48.6)	1410 (55.4)	
**ACAM2000^®^ MP**	179 (51.4)	1136 (44.6)	**0.01**
**Rates Per 100,000**			
**Dryvax^®^ Rate (95% CI)**	12.1 (10.4–14.1)		
**ACAM2000^®^ Rate (95% CI)**	15.9 (13.7–18.4)		**0.01**
**Age in years: Median (IQR) Range**	24 (21, 30) 18–57	23 (21, 30) 17–73	
**Age Categories**			
18–24	183 (52.6)	1449 (56.9)	
25–29	76 (21.8)	453 (17.8)	
30–39	76 (21.8)	442 (17.3)	
> = 40	13 (3.8)	203 (8.0)	**0.001**
**Gender**			
Male	334 (96.0)	2236 (87.8)	
Female	14 (4.0)	311 (12.2)	
Unknown	0	1 (0.0)	**<0.0001**
**Race**			
White	287 (82.5)	1630 (64.0)	
Black	36 (10.4)	383 (15.0)	
Other	5 (1.4)	181 (7.1)	
Not specified	20 (5.7)	352 (13.9)	**<0.0001**

The MP cohort associated with ACAM2000^®^ in the context of background vaccination frequency (51.4%) were more frequent in the registry compared with the older Dryvax^®^ vaccine (48.6%) with a difference of 6.8% (95% CI: 1.6, 12.0). The rates of documented MP VAERS cases (including 4 deaths) from the background immunized population were 12.1 (95% CI 10.4,14.1) per 100,000 Dryvax^®^ recipients and 15.9 (95% CI: 13.7,18.4) per 100,000 ACAM2000^®^ recipients (Table 3).

Demographics and acute clinical symptoms (cardiac and systemic) in the MP cohort with a comparison between myocarditis and pericarditis (Table 4) demonstrated that the majority of myocarditis cases had chest pain (96.7%) with dyspnea and/or fatigue alone in 3.3%. All the pericarditis cases had chest pain or chest pain variants with acute presentation. The only significant differences were a greater percentage of females in the pericarditis group by 12.4% (95% CI: 6.0,22.4) and a greater frequency of positional/movement associated pain exacerbation by 30.9% (95% CI: 20.2,39.2). However, 58% of the myocarditis cohort also described positional/movement association of pain exacerbation.

**Table 4:** Comparison of myocarditis and pericarditis clinical characteristics.

Myocarditis/Pericarditis Cases	All Cases N (%)	Myocarditis N (%)	Pericarditis N (%)	P Value M & P
**Case Numbers**	**348**	**276**	**72**	
**Age in years**: Median (IQR) Range	24 (21,30) 18–57	24 (21,29) 18–55	26 (22,33.5) 19–57	**0.03**
**Gender**				
Male	334 (96.0)	272 (98.6)	62 (86.1)	
Female	14 (4.0)	4 (1.4)	10 (13.9)	**<0.0001**
**Time (Days) to Onset**				
Cardiac Symptoms: Median (IQR) Range	10.0 (8,11) 1–35	10 (9,11) 1–29	9 (7,11.5) 2–35	0.05
Diagnosis: Median (IQR) Range	11.0 (10,12) 1–48	11 (10,12) 1–29	10.5 (9,13) 4–48	0.57
**Clinical Presentation**	**348**	**276**	**72**	
Chest pain	339 (97.4)	267 (96.7)	72 (100.0)	0.21
Dyspnea	237 (68.1)	184 (66.7)	53 (73.6)	0.26
Palpitations	64 (18.4)	48 (17.4)	16 (22.2)	0.35
Edema	2 (0.6)	0 (0.0)	2 (2.78)	0.04
Nausea	73 (21.0)	63 (22.8)	10 (13.9)	0.10
Positional/Movement Pain ↑	224 (64.4)	160 (58.0)	64 (88.9)	**<0.0001**
**Cardiac Symptoms**:				
2 or more	264 (75.9)	205 (74.3)	59 (81.9)	
< 2	84 (24.1)	71 (25.7)	13 (18.1)	0.18
**Systemic Symptoms**				
Fever/Chills	156 (44.8)	130 (47.1)	26 (36.1)	0.09
Diaphoresis	76 (21.8)	63 (22.8)	13 (18.1)	0.38
Headache	101 (29.0)	79 (28.6)	22 (30.6)	0.75
Myalgias/muscle aches	107 (30.7)	89 (32.3)	18 (25.0)	0.24
Fatigue	123 (35.3)	98 (35.5)	25 (34.7)	0.90
GI (nausea, vomiting, diarrhea, abdominal pain)	48 (13.8)	43 (15.6)	5 (6.9)	0.06
Upper respiratory symptoms	11 (3.2)	9 (3.3)	2 (2.8)	1.00
**Systemic symptoms**:				
2 or more	186 (53.4)	154 (55.8)	32 (44.4)	
1	74 (21.3)	58 (21.0)	16 (22.2)	
None	88 (25.3)	64 (23.2)	24 (33.4)	0.15

Other systemic symptoms included fever/chills (44.8%), diaphoresis (21.8%), headaches (29.0%), myalgias/muscle aches (30.7%), fatigue (35.3%), and gastrointestinal symptoms (13.8%) with two or more systemic symptoms in 53.4%. There were no significant differences in symptom patterns between the myocarditis and pericarditis cohorts. While the majority of case records did not describe a detailed cardiac examination, 15 cases were identified as having a friction rub (information regarding 3-component detail not provided) with a breakdown of 12/276 (4.3%) myocarditis and 3/72 (4.1%) pericarditis cases. Most pericarditis cases had no documentation of a pericardial friction rub (pertinent positives or negatives).

Cardiac diagnostic test results in MP and by myocarditis versus pericarditis cohorts (Table 5) included abnormal electrocardiograms (61.9%) with significantly less frequent diagnostic changes in pericarditis by a difference of 30.5% (95% CI: 17.4,42.4). Pericardial effusion was noted in less than 10% of both MP cohorts with no significant differences. Inflammatory marker data (when available) showed elevations in both cohorts (92.4% and 84.6%).

**Table 5:** Frequencies of abnormal cardiac tests by diagnosis (Myocarditis and pericarditis).

Myocarditis/Pericarditis Case Review (%)	All Cases 348	Myocarditis 276	Pericarditis 72	P Value
**Diagnostic Data**				
Electrocardiogram (ECG) *Performed/Data Available	**336**	**267**	**69**	
ECG Abnormal: N (% of Total Data Available)	208 (61.9)	182 (68.2)	26 (37.7)	**<0.0001**
**Inflammatory Marker**	**144**	**118**	**26**	
CRP &/or ESR Elevation	131 (91.0)	109 (92.4)	22 (84.6)	0.25
**Cardiac Injury Measures**				
Any Cardiac Enzyme Test	**335**	**276**	**59**	
Any Confirmed Cardiac Enzyme Elevation	284 (84.8)	268 (97.1)	16 (27.1)	**< 0.0001**
CKMB Data Available	**251**	**211**	**40**	
CKMB Abnormal	175 (70.0)	170 (80.6)	5 (12.5)	**< 0.0001**
Troponin I/T/unspecified	**333**	**275**	**58**	
Troponin Positive	282 (84.7)	270 (98.2)	12 (20.7)	**<0.0001**
**Cardiac Imaging Data**	**322**	**265**	**57**	
Ejection fraction (EF) ≥60%	148 (46.0)	116 (43.8)	32 (56.1)	0.09
EF normal (>55%)	222 (68.9)	174 (65.7)	48 (84.2)	
EF low normal (51–55%)	53 (16.5)	47 (17.7)	6 (10.5)	
EF ≤ 50% (Range: 19–50)	47 (14.6)	44 (16.6)	3 (5.3)	**0.02**
Hypokinesis	52 (16.1)	48 (18.1)	4 (7.0)	**0.04**
Abnormal: EF≤55% &/or Hypokinesis	114 (35.4)	104 (39.2)	10 (17.5)	**0.002**
Pericardial Effusion	26 (8.1)	21 (7.9)	5 (8.8)	0.79
**Coronary Artery (CA) Test**	**92**	**85**	**7**	
Non-obstructive disease	7 (7.6)	7 (8.2)	0 (0.0)	1.00
**CMR Done/Data Available**	**69**	**59**	**10**	
Abnormal CMR	32 (46.4)	27 (45.8)	5 (50.0)	1.00

Qualitative or quantitative documentation of any cardiac specific enzyme elevations (CKMB and/or cardiac troponin; Table 5) was predominantly in the myocarditis cohort (97.1%). The cardiac enzyme predominantly measured acutely was troponin (I or T or not specified) with quantitative levels (if available) greater than 0.10 ng/ml in 90.1% of myocarditis cases. Measurable troponin interpreted as “normal range” acutely was variably documented in 12/58 pericarditis cases but was less than 0.10 ng/mL.

Imaging studies providing left ventricular ejection fraction data were predominantly normal (EF>55%) in the entire cohort (68.9%) but with a higher percentage in pericarditis cases (84.2%). Hypokinesis and/or EF≤55% were present in 39.2% of myocarditis versus 17.5% of pericarditis patients (Table 5), a difference of 21.7% (95% CI: 8.7,31.8). S3 Table provides expanded information about ejection fraction stratification and associated documentation of hypokinesis.

Coronary angiography was performed in 85/276 (30.8%) myocarditis patients, with 7/85 (8.2%) having abnormal findings limited to non-obstructive coronary artery disease (Table 5). Only 7/72 (9.7%) pericarditis patients had coronary angiography, with none showing coronary artery disease. CMR in 27/59 (45.8%) myocarditis patients demonstrated focal or diffuse myocardial edema and/or damage, including late gadolinium enhancement (Table 5). Abnormal CMR findings in pericarditis patients were predominantly pericardial effusions (Table 5).

Patterns of medications prescribed for acute myocarditis or pericarditis (S4 Table) were comparable except for initial treatments consistent with possible myocardial infarction in those patients with a final diagnosis of myocarditis (18.4% versus 7.3%), a difference of 11.1% (95% CI: 1.7,17.9). About 86.0% of MP patients received acute treatment with non-steroidal anti-inflammatory drugs, including aspirin, with no difference between MP cohorts. Aspirin was predominantly prescribed for patients diagnosed with myocarditis (25.8%) rather than pericarditis (8.7%), a difference of 17.2% (95% CI: 7.1,24.7). Other non-steroidal therapies were prescribed for 73.9% of patients with pericarditis versus 60.3% with myocarditis, a difference of 13.6% (95% CI: 0.9,24.5). Other therapies with no differences between MP cohorts included colchicine (16.4%), narcotics (16.4%), and gastrointestinal symptom treatment (9.2%).

One or more cardiovascular disease (CVD) risk factors (S4 Table) were present in 28.2%, with no significant differences in frequency between MP cohorts. The major factors were dyslipidemia (12.9%) and/or family history of early CVD major events (15.5%). It is noteworthy that the smallpox vaccine program exempted individuals with three or more CVD risk factors from vaccination. While BMI equal to or greater than 30% was present in 17.5%, there are no data available to verify those with inaccurate calculated BMI from actual body fat as detailed under methodology. Tobacco use was described in 38.2%.

There were no significant gender differences in the clinical presentation and medications prescribed for MP patients (S5 Table). However, out of 14 female patients, 71.4% were diagnosed with pericarditis compared to 18.6% for male patients (62/334), with a difference of 52.8% (95% CI: 26.4, 70.4).

### Recovery

From the MP cohort (348), 306 patients had follow-up data available with full recovery in 87.3% (Table 6). The recovery rate by vaccine type (Dryvax^®^ versus ACAM2000^®^) was not significantly different: 88.7% (126/142) versus 86.0% (141/164). There was no significant difference in recovery rates between myocarditis (87.1%) and pericarditis (87.7%) patients. The majority of myocarditis (76.7%) and pericarditis (68.4%) patients recovered in less than one year, with median times for both less than six months (Table 6). There was also no significant difference in the percentage of those who recovered in <1 year by vaccine type.

**Table 6:** Comparison of rates and timeline (years) of recovery after SPV myocarditis versus pericarditis with associated measures of cardiac injury at baseline.

Myocarditis-Pericarditis Outcomes	MP (%)	Myocarditis (%)	Pericarditis (%)	P Value
**MP Cases**	**348**	**276**	**72**	
**Follow-up data**	**306 (87.9)**	**241 (87.3)**	**65 (90.3)**	
Recovered Cases	267 (87.3)	210 (87.1)	57 (87.7)	0.91
In ≤ 6 weeks	16 (5.2)	7 (2.9)	9 (13.8)	**0.002**
In <1 vs ≥1 year				
Recovered < 1 year	200 (74.9)	161 (76.7)	39 (68.4)	
Recovered ≥ 1 year	67 (25.1)	49 (23.3)	18 (31.6)	0.20
**Time to Recovered**				
Median years (IQR) Range	0.36 (0.19,1.03) 0.11–16.46	0.35 (0.19,0.84) 0.11–16.46	0.41 (0.15,1.98) 0.11–7.91	0.89
**Recovery in < 1 Year**	**200**	**161**	**39**	
Median years (IQR) Range	0.25 (0.16,0.45) 0.11–0.98	0.25 (0.18,0.45) 0.11–0.95	0.19 (0.12,0.45) 0.11–0.98	0.06
**Recovery ≥ 1 Year**	**67**	**49**	**18**	
Median years (IQR) Range	3.15 (1.44,5.74) 1.03–16.46	3.06 (1.30,7.20) 1.03–16.46	3.48 (2.17,4.59) 1.42–7.91	0.60

**Fig 2:**
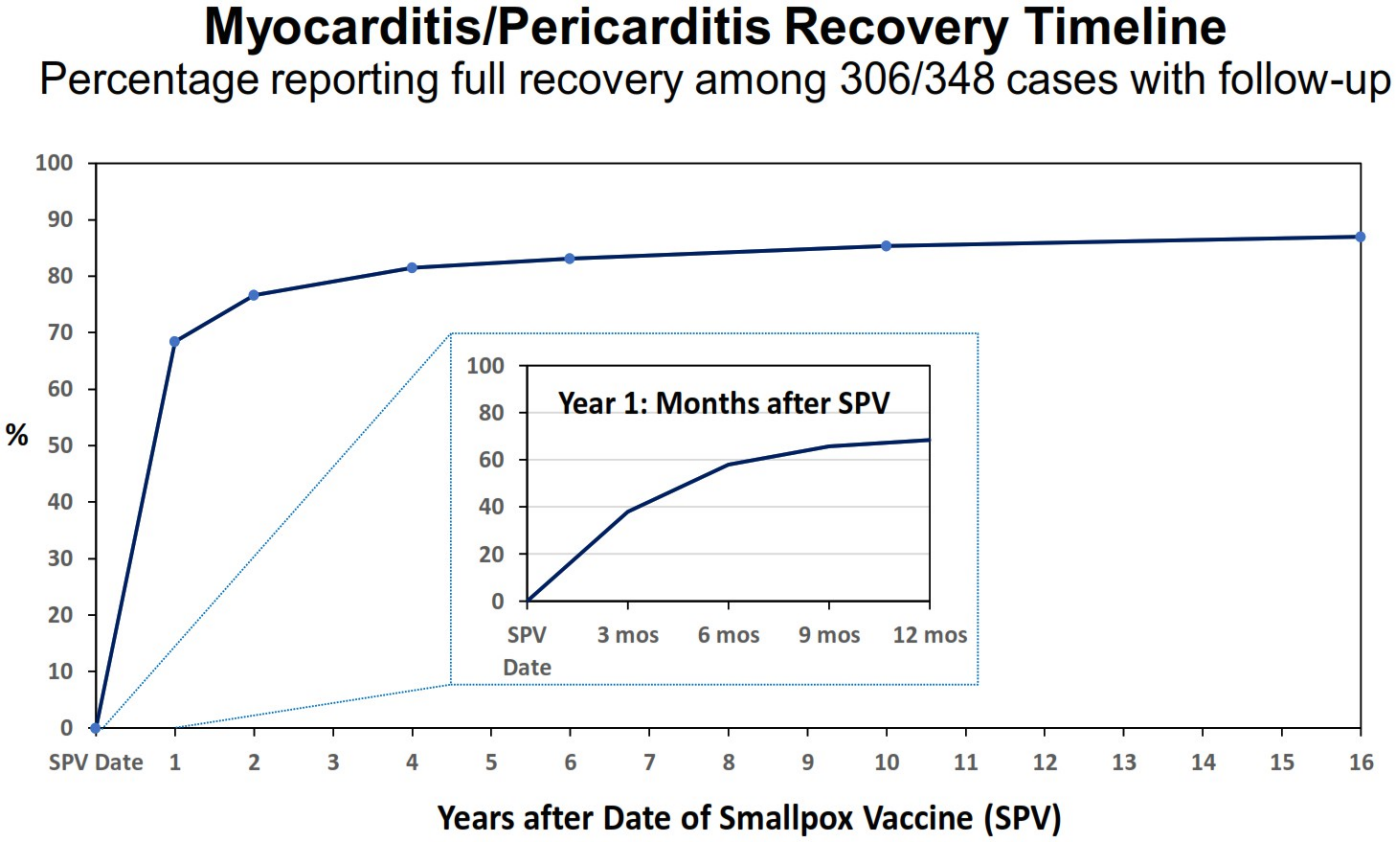
Timeline of myocarditis/pericarditis case recovery percent after date of vaccination.

MP patient recovery within 6 weeks after the vaccination date represented 5.2% of the cohort (2.9% myocarditis and 13.8% pericarditis with a percent difference of 10.9% and 95% CI: 4.0,21.5). However, 25.1% of MP patients that recovered beyond one year had a median time of 3.15 years (IQR 1.44, 5.74; range 1.03,16.46 years). MP patients with partial or no documented recovery by the last date available (beyond one year) represented 12.7% (39/306) of the cohort. (Table 6) Fig 2 provides a graphical timeline of percent recovery by time after the date of vaccination. The breakdown by myocarditis and pericarditis recovery at each year point is presented in S1 Fig.

Comparisons between MP cohorts that recovered in less than one year versus one year or longer (or no documented full recovery) show comparable demographics (age, race, gender) as well as baseline rates of positive troponins and quantitative troponin levels greater than or equal to 0.100 ng/mL (Table 7). Duration of follow-up for the MP cohort (n = 306) was 3.1 years (median) with interquartile range of 1.27 to 8.4 years (range of 0.13, 18.38).

**Table 7:** Comparison of MP cases by recovery rates (less than one year versus prolonged or no recovery after one year or longer) for baseline measures of cardiac injury, function, cardiac risk factors, and acute treatment.

Myocarditis & Pericarditis (%)	All Cases	Recovered/No Symptoms < 1-year	Prolonged ≥ 1 year or No Recovery	P Value
**Case Numbers with Follow-up Data**	**306**	**200**	**106**	
Myocarditis/Pericarditis	241/65	161/39	80/26	
**Time to Recovery (Years)**	**267 (87.3%)**	**200**	**67**	
Median (IQR) Range	0.36 (0.19,1.03) 0.11–16.46	0.25 (0.16,0.45) 0.11–0.98	3.15 (1.44,5.74) 1.03–16.46)	
**Baseline Cardiac Injury**				
Troponin Qualitative (All)	**292**	**190**	**102**	
Troponin Positive	247 (84.6)	163 (85.8)	84 (82.4)	0.44
Troponin Quantitative	**225**	**152**	**73**	
Troponin < 0.100 ng/mL	17 (7.6)	8 (5.3)	9 (12.3)	
Troponin ≥ 0.100 ng/mL	208 (92.4)	144 (94.7)	64 (87.7)	0.06
**Cardiac Imaging**				
EF>50% versus ≤50%	**283**	**184**	**99**	
EF > 50%	239 (84.5)	163 (88.6)	76 (76.8)	
EF ≤ 50%	44 (15.5)	21 (11.4)	23 (23.2)	**0.01**
**Imaging of Wall Motion**				
Hypokinesis	50 (17.7)	26 (14.1)	24 (24.2)	**0.03**
**Pericardial Imaging**				
Pericardial Effusion	21 (7.4)	16 (8.7)	5 (5.1)	0.26
**Cardiac Risk Factors**	**306**	**200**	**106**	
None (0)	219 (71.6)	144 (72.0)	75 (70.8)	
1 or more	87 (28.4)	56 (28.0)	31 (29.2)	0.82
**Acute Medications**	**296**	**193**	**103**	
NSAID therapy	187 (63.2)	113 (58.5)	74 (71.8)	**0.02**
Corticosteroid Therapy	6 (2.0)	3 (1.6)	3 (2.9)	0.42

When comparing presenting rates of ejection fraction ≤50% versus >50%, the early recovery versus long/no recovery rates were 11.4% versus 23.2% with a difference of 11.8% (95% CI: 2.8, 21.9). Hypokinesis was present in cases with prolonged recovery more frequently than in those who took less than a year, with a difference of 10.1% (95% CI: 0.8, 20.5). Hypokinesis was documented in 50/283 MP cases (Table 7), but EF was normal (>55%) in 14, low normal (51–55%) in 9, and abnormal (≤50%) in 29. Both hypokinesis and an EF≤50% were associated with prolonged (>1 year) recovery or no recovery by the last follow-up date.

The significant findings in Table 7 represent the myocarditis cohort (further documented in Table 8). It is noteworthy that 44.2% (102/231) of the myocarditis patients had presenting EF ≥60%, with 79.7% (94/118) of recovered patients in this range. An EF increase of 8% or greater (from acute to recovered status) was seen in 39.5% of myocarditis patients (Table 8), with presenting or recovered EF greater than 55% in most cases.

**Table 8:** Comparison of Myocarditis cases by recovery rates (less than 1 year versus prolonged or no recovery after 1 year or longer) for baseline measures of cardiac injury, function, cardiac risk factors, and acute treatment.

Myocarditis Cohort (%)	All Cases	Recovered <1-year	Prolonged or No Recovery	P Value
**Myocarditis 241/306**	**241**	**161**	**80**	
**Recovery Time in Years**	**210**	**161**	**49**	
Median (IQR) Range	0.35 (0.19,0.84) 0.11–16.46	0.25 (0.18,0.45) 0.11–0.95	3.06 (1.30,7.20) 1.03–16.46	
**Baseline Cardiac Injury**				
Troponin Qualitative	**240**	**161**	**79**	
Acute troponin POS	235 (97.9)	158 (98.1)	77 (97.5)	0.67
Troponin Quantitative	**217**	**148**	**69**	
Troponin < 0.100	12 (5.5)	5 (3.4)	7 (10.1)	
Troponin ≥ 0.100 ng/ml	205 (94.5)	143 (96.6)	62 (89.9)	0.06
**Cardiac Function**				
EF>50% versus ≤50%	**231**	**155**	**76**	
EF > 50%	190 (82.3)	134 (86.5)	56 (73.7)	
EF ≤ 50%	41 (17.7)	21 (13.5)	20 (26.3)	**0.02**
**EF ≥60% & Changes**				
EF ≥60% at baseline	102/231 (44.2)	69/155 (44.5)	33/76 (43.4)	0.87
EF ≥60% after recovery	94/118 (79.7)	58/71 (81.7)	36/47 (76.6)	0.50
EF increased ≥8%	45/114 (39.5)	24/69 (34.8)	21/45 (46.7)	0.20
**Imaging of Wall Motion**				
Hypokinesis	46 (19.9)	24 (15.5)	22 (28.9)	**0.02**
**Pericardial Imaging**				
Pericardial Effusion	16 (6.9)	12 (7.7)	4 (5.3)	0.49
Treatment		**<1-year**	**Prolonged**	
With NSAID	140 (59.8)	86 (54.8)	54 (70.1)	
Without NSAID	94 (40.2)	71 (45.2)	23 (29.9)	**0.02**
EF≤50% with NSAID	20 (50.0)	7 (35.0)	13 (65.0)	
EF≤50% without NSAID	20 (50.0)	13 (65.0)	7 (35.0)	0.06
Corticosteroid Therapy	5/233 (2.1)	2/156 (1.3)	3/77 (3.9)	0.34

Among patients with myocarditis, the percentage who had a delayed time to recovery at time of the last follow-up was 12.8% (95% CI: 2.1,24.7) higher in those with an acute left ventricular ejection fraction (EF) of ≤ 50% and 13.5% (95% CI: 2.4,25.7) higher in those with hypokinesis compared to patients without these findings. Non-steroidal anti-inflammatory therapy use showed a higher percentage of cases in the prolonged recovery versus rapid recovery cohorts with a difference of 15.3% (95% CI: 2.0, 27.6) (Table 8). However, the difference for EF ≤50% with NSAID and without was not significant by recovery time. The majority of myocarditis patients had been treated with NSAID, including those with acute EF ≤50% in both the short recovery (7/20; <1-year) and prolonged recovery (13/20; ≥1 year) groups (Table 8). There was no difference in frequencies for pericarditis (S6 Table).

Recurrent cardiac symptoms impacting the patient perception of recovery were present in 39.8% of myocarditis (n = 241), with 14.3% (23/161) in the short recovery cohort, 89.8% (44/49) in the prolonged recovery subset and 93.5% (29/31) in the no recovery subset with extended follow-up. Other recurrent/persistent symptoms included chronic fatigue, reduced exercise tolerance, and dysautonomia-like symptoms. Atrial arrhythmias were documented in 11 myocarditis (2 required radiofrequency ablations) and 3 pericarditis patients. Ventricular arrhythmias were noted in 4 myocarditis patients (2 required indwelling cardiac defibrillators) and 2 in the pericarditis cohort (without impacting symptoms). Six myocarditis patients were diagnosed with cardiomyopathy; three had a prolonged recovery, and three remained symptomatic at the last available follow-up (>5 years). Two patients initially classified as pericarditis received delayed diagnoses of cardiomyopathy with no recovery at last follow-up date and no records with cardiac function data available. A pericardial window was required for pericardial effusion in 1 pericarditis patient. One myocarditis patient who presented acutely with a cardiac arrest requiring resuscitation had a full recovery by one year of follow-up. Another myocarditis patient with prolonged symptoms and normalized cardiac function experienced an acute flare of myocarditis in temporal association with an annual influenza vaccine seven years later. This patient ultimately fully recovered by the fifth year following the second acute event.

### Death cases

The four adjudicated MP deaths (Table 9) included two sudden deaths (with evidence for hypersensitivity eosinophilic myocarditis and no other causes (**Case 1** and **2**). **Case 3** had a final causality assessment (after extensive additional testing and review) of contributory SPV myocarditis with arrhythmia and cardioversion (**Case 3**). The fourth case was female with autopsy findings of epicarditis and autoimmune disease associated with positive antinuclear antibodies (SSA/Ro positive).

**Table 9:** Characteristics of four acute deaths with evidence of myocarditis or pericarditis following SPV.

Case Number	1	2	3	4
**Age**	26	24	18	23
**Gender**	Male	Male	Male	Female
**Race**	White	White	Black, Other	Black
**BMI>30, calculated**	No	Yes	No	No
**Cardiac Risk Factors #/6**	0	1	0	0
**Other Vaccines # (type)**	1 (Influenza, Split)	3 (Typhoid Vi, HPV)	2 (Typhoid Vi, ANTH)	4 (ANTH1, HepB2, MMR, Typhoid Vi)
**Days from Vaccine to Symptom onset**	10	12	8	10
**Days: Vaccine to Death**	16	12	13	32
**Dryvax^®^ or ACAM2000^®^**	Dryvax^®^	ACAM2000^®^	ACAM2000^®^	Dryvax^®^
**Vaccine Reaction, Initial**	Fever, chills, malaise, adenopathy, NSAID	Unknown, did not seek care post vaccine. Allergic rhinitis antihistamine	Fatigue and increased thirst in AM prior to acute arrhythmia during 5-mile run	Fatigue, Left shoulder pain, fever, chills, shortness of breath, NSAID
**Special issues**	Sudden death	Sudden death	Resuscitated; Complications during 5-day hospitalization	Prolonged illness; Pleural effusion; Cardiac arrest
**Peak Troponin ng/mL**	—	—	I: 1.310	I: <0.04 (non-acute)
**Hospitalized**	No	Yes	Yes	Yes
**Arrhythmia documented**	No	No	Yes	No
**Toxicology**	Negative	Negative	Negative	Negative
**Histology at Autopsy**	Myocarditis (eosinophils, mixed lymphocytic)	Necrotizing Eosinophilic Myocarditis Mild concentric hypertrophy	Histology due to prolonged ischemia, final diagnosis attributed events to SPV myocarditis	Epicarditis with pre-event asymptomatic positive ANA, SSA/Ro (4)
**Viral Studies (PCR Heart tissue, Vaccinia included)**	Negative	Negative	Negative	Negative

In all four cases, baseline health (prior to immunization) was reviewed in medical records documenting good health, meeting military physical fitness standards, and without diagnoses impacting the ability to deploy or receive the SPV. Coronary artery disease or cardiomyopathy was not present in any of the cases. Concomitant vaccines administered with the SPV varied in number (1–4) and included the following: Typhoid Vi, Human papillomavirus (HPV), anthrax (ANTH), Hepatitis B (HepB), measles, mumps, rubella (MMR). Final causality assessments relied heavily on histology at autopsy, clinical history, and absence of other causes. Each case had extensive negative viral PCR studies (including vaccinia of the heart tissue). **Cases 1 and 2** had confirmed evidence of hypersensitivity myocarditis with no other cause. **Case 3** was previously reported as a case of rhabdomyolysis based on peak creatine phosphokinase values approaching 200,000. The initial autopsy reported finding no evidence of myocarditis [25]. However, an extensive multidisciplinary clinical review with additional testing subsequently resulted in a causality assessment that recurrent supraventricular arrythmia secondary to SPV-associated probable myocarditis was likely a significant contributor to the initiating presentation and eventual outcome. Supplemental information about this case is included in S7 Table.

**Case 4** was a young, healthy, physically active, and asymptomatic female with new onset prolonged systemic symptoms and evolving dyspnea following SP primary immunization. She was diagnosed with autoimmune disease (positive antinuclear, anti-SSA/Ro, and cardiac autoantibodies) and died of a cardiac arrest in the hospital. The autoantibodies were present in stored sera during an asymptomatic time two years before the acute event. Causality was attributed to autoimmune activation by immune inflammation associated with the primary SPV and epicarditis at autopsy.

For military smallpox vaccinees, the overall death rate was 2 per million (95% CI: 0.42,4.0).

## Discussion

The current report provides acute presentation and follow-up recovery data on the largest hypersensitivity myocarditis and pericarditis cohort linked to the vaccinia smallpox vaccine in the context of over 2.5 million service members immunized over 14 years. In contrast to other reports related to eosinophilic myocarditis, the experience with SPV-associated hypersensitivity myocarditis demonstrates a trajectory of full recovery in 87.1% of cases, with 76.7% recovered in the first year after vaccination (Table 7). Over 50% healed “back to baseline health” and frequently returned to complete pre-event activity levels in less than six months, particularly with pericarditis or rapid recovery from “mild” myocarditis similar to the 2020 report by Patriki et al. [26].

The four deaths detailed during the 2002–2016 period represent very rare cases, with only two having a sudden death associated with classic hypersensitivity myocarditis histology. The challenge of causality assessments for complex, prolonged illness following acute presentation before death often involves divergent opinions depending on specialty perspectives within a multidisciplinary review. In addition, prolonged resuscitation can obscure classic myocarditis histology due to over-riding ischemic injury (forensic pathology communications). The ability to actively investigate cases with direct verification of history with family members, associates, and attending healthcare workers enhanced the precision of data available for this review. In addition, it enabled compassionate support of surviving family, friends, and co-workers with many questions and a need to process their grief.

Of the 106 with more than one year of follow-up and ongoing symptoms, 63% recovered with a median duration of 3.2 years (IQR 1.4,5.7). Of those MP cases with acute reduced EF (≤50%: 44/283: 15.5%) and/or hypokinesis (50/283: 17.7%), recovery in less than one year occurred in 47.7% and 52.0%, respectively (Table 7). Our findings draw attention to potential long-term morbidity and possible chronic (>3 months) or recurrent (return after a quiescent interval of 4–6 weeks) symptoms in pericarditis and myocarditis [26, 27]. Ventricular function and cardiac troponins were usually normal in those with prolonged or recurrent cardiac symptoms, and a specific diagnosis was challenging to confirm, especially since CMR was infrequently completed. The observation of an 8% (or more) increase in EF during recovery among those with presumed “normal” EF during acute evaluation has implications for acute clinical management, particularly in young, healthy, physically active individuals whose actual normal EF may be greater than 60%. The exact mechanism of chronic MP symptoms is not well understood and may be heterogeneous. Some individuals may have a genetic predisposition and may develop autoantibodies as suggested with MP following COVID-19 mRNA (messenger RNA) vaccines [28]. Our findings that 25.1% of patients with long-term follow-up experienced symptoms beyond 12 months after the acute event mirror reports in the literature of chronic and/or recurrent pericarditis symptoms with normal cardiac enzymes [27].

Non-steroidal anti-inflammatory drugs were used to treat cardiac chest pain symptoms in 59.8% of myocarditis cases, including 50% of those with depressed ejection fraction (≤50%). There were no documented reports of adverse effects related to NSAID. However, given the limitations of a retrospective and observational study design, we cannot infer definite conclusions about the safety of NSAID use in hypersensitivity myocarditis. A 2019 retrospective case-controlled study with a 12-month follow-up demonstrated the safety of NSAID in myocarditis with preserved EF. However, the study authors recommended a randomized prospective study to address the question definitively [29].

Early reviews of hypersensitivity myocarditis focused on eosinophilic myocarditis, often described with peripheral eosinophilia, drug rashes, and vasculitis features with a poor prognosis and mortality risk, particularly if associated with hypereosinophilic syndromes or without prompt corticosteroid therapy [30–33]. Hypersensitivity myocarditis associated with drugs or vaccines presents at the lower end of the spectrum of clinical severity with potentially differing long-term outcomes depending on the specific drug or vaccine and systemic disease associations [34]. The subset of hypersensitivity myocarditis cases associated with vaccines, often without systemic elevations in eosinophil counts, is frequently associated with recovery and rapid response to corticosteroids [14, 31–34]. In contrast with hypereosinophilic syndrome cardiomyopathies, smallpox vaccine hypersensitivity myocarditis cases had a very rare mortality risk [25, 35].

Before the twentieth-century experience with the smallpox vaccine in young adults, drug or vaccine-associated hypersensitivity myocarditis was considered a rare subset of non-infectious myocarditis temporally linked to a possible drug exposure and rarely reported [5, 7, 17, 35]. Primarily in case reports, histologic findings demonstrated inflammatory infiltrate rich in eosinophils and no evidence of a pathogen [31–35]. Documentation of a possible association of SPV with myocarditis was not described in any reports of SPV adverse events based on prior efforts of large vaccination programs nor in publications of 2003 [36, 37]. Subsequent epidemiologic surveillance in the CDC civilian and DOD programs reported rates per 100,000 of 51.9 and 11.7, respectively [5, 7, 38]. Prospective studies associated with monitoring of electrocardiograms and biomarkers of cardiac injury documented much higher rates per 100,000 (78.3–345.6) compared to passive or enhanced surveillance approaches [17, 25, 39]. These reports highlight the importance of enhanced active surveillance and programmatic investment in surveillance infrastructure (including clinical care consultation resources with adverse drug/vaccine reaction expertise to include adversomics) as well as the need for prospective studies [5, 7, 9, 17, 25, 39].

The VAERS rate per 100,000 vaccinees in this report (13.8; 95% CI: 12.4,15.3) is comparable to the 2021 report for ACAM2000 (15.8; 95% CI 13.3,18.7) [25]. As documented in the prospective studies, retrospective surveillance underestimates the true incidence and prevalence of adverse events. In addition, two prospective studies documented evidence of possible subclinical myocarditis by cardiac enzyme elevations alone (316 and 2867 per 100,000) during the post-SPV time window associated with inflammatory cytokine elevations in first dose vaccinees [17, 39, 40]. The difference in rates can be attributed to troponin assay sensitivity [41] variability and the fact that pre-post paired sample testing was used in the 2015 prospective study [17] but not in the 2020 report [39]. The clinical significance of these observations and the associated long-term prognosis remains undefined.

The 2022 report from the World Health Organization Global Surveillance Pharmacovigilance Database with individual case safety reports from 1967 through 2020 detailed the experience with myocarditis reports associated with drugs and vaccines from 47 countries. [35] With over 6000 suspected drug-induced cases, 5108 suspected myocarditis cases are associated with 62 drugs, and 41 of these are clustered in 5 classes, including antipsychotics, salicylates, anti-neoplastic-cytotoxic, immunotherapies and vaccines (790, 15.5%) (S8 Table). The most represented drugs are clozapine, immune checkpoint inhibitors, mesalazine (NSAID, also known as mesalamine), and the smallpox vaccine (383/5108). Other vaccines associated with myocarditis reports are influenza, anthrax, diphtheria, tetanus, pertussis (with or without polio), hepatitis A and/or hepatitis B, Typhoid, meningococcal, Tick-borne encephalitis, and Japanese encephalitis. However, many anthrax vaccine reports included SPV exposure simultaneously (S8 Table). In addition, this report reflected data from before the COVID-19 vaccines.

Like the smallpox vaccine safety surveillance experience, early reports regarding COVID-19 vaccine-associated adverse events did not identify a myocarditis/pericarditis signal. The active surveillance application (V-safe) did not include chest pain as a selectable symptom [42, 43]. The MHS platform of active VAERS patient support and care facilitation (IHD) first verified the MP signal in young, predominantly white males [44]. It is noteworthy that the MHS (IHD) was able to rapidly identify this MP signal because of the lessons learned from the enhanced smallpox vaccine safety surveillance that reinforced the culture of looking for and reporting new-onset cardiac symptoms. Subsequent reports validated the actual signal of myocarditis and pericarditis following COVID-19 vaccines [45–49]. Demographics, morbidity and mortality, and recovery timelines appear similar to cases described in this study of live virus vaccinia recipients [50, 51]. However, long-term outcomes remain to be defined and may need to include prolonged stress reactions associated with an acute case of MP in a previously healthy and fit vaccinee.

## Conclusion

Hypersensitivity myocarditis and pericarditis following the smallpox vaccine are associated with complete clinical and functional ventricular recovery in over 87% of cases (74.9% <1 year). A minority of MP cases experienced prolonged or incomplete recovery beyond one year.

## Limitations of study

Retrospective observational study design limitations include variable frequency and duration of follow up as well as variable data quality and availability. Identifying clinical cases that met the case definition was limited by where care was provided and if the initial evaluation arrived at the proper diagnosis with consideration for reporting as an adverse event. Even with classic acute onset cardiac symptoms, some patients reported that the emergency department considered them too young to have a cardiac diagnosis [52]. Since VAERS is a passive surveillance system, it is well recognized as underestimating the true incidence of disease with a bias for detecting severe cases that result in emergency room care and hospitalization [53]. Despite the limitations in passive vaccine safety surveillance, this report (which includes case ascertainment from a proactive stimulated reporting process) includes a large enough cohort of patients to enable the primary study aims of natural history to be achieved.

The limited availability and use of cardiac magnetic resonance within this patient cohort, in contrast to current recommendations [54], leaves open questions about potential subclinical inflammation that might have been detected with high-sensitivity cardiac troponin levels. While cardiac-specific troponin assays are considered sensitive and specific for myocardial injury, the testing can be associated with false positive and negative results, including improper blood sample handling or anti-troponin antibodies [41, 55].

The limitations of retrospective reviews of deaths occurring in temporal association with immunization make vaccine causality determination inherently challenging. While the consistency of findings, the strength of association, biologic plausibility, and temporal relationship with vaccine receipt are factors considered in the absence of other causes. However, underlying comorbidity and other signs and symptoms surrounding the acute event precipitating death impact the level of certainty for causal associations [56].

## Future directions

Improving our understanding of myocarditis and pericarditis as adverse events after vaccines has become an evolving need, as highlighted by the global attention to the association with messenger RNA Covid-19 vaccines [44–49]. The smallpox vaccine [5, 7, 9, 17, 25, 39] as well as the COVID-19 vaccine [44, 46] experiences with identifying epidemiologic and prospective evidence of a causal association with myocarditis and pericarditis, may inform future efforts to improve diagnosis, treatment, and prevention of this type of adverse event.

Of particular interest is the fact that some of the patients included in our report had serologic evidence of cardiac-specific autoantibodies (myosin and beta-adrenergic receptor specificity) before the vaccinia exposure raising questions about a potential marker of risk for hypersensitivity MP [57]. The two patients who developed ventricular arrhythmias and several of the death cases had evidence of these autoantibodies in sera available before the vaccination date. Further studies are needed to address risk factors for development of this immune mediated reaction to include potential evaluation of pre and post vaccine sera to determine if the vaccine associated immune response triggers a quantitative or qualitative change in cardiac specific autoantibodies (further supporting a hypersensitivity-autoimmune mechanism) [32]. Anti-inflammatory therapy and immune modulation strategies may improve drug- or vaccine-associated hypersensitivity myocarditis recovery.

Given the concerns about long-term morbidity and mortality with vaccine-associated MP, there is a need for ongoing collaborative support for multi-center clinical registries connected with the patients directly to ensure accurate data regarding patient outcomes and to capture lessons learned regarding optimum follow-up care and therapeutic interventions. Bias in adverse event case detection is often related to insufficient data to classify a case diagnosis. There is an underestimation of case detection as shown by the difference in case level of certainty between passive surveillance (CDC VAERS) and enhanced case surveillance with clinical outcomes monitoring over time (MHS), as detailed by McMahon et al. [9]. In addition, long term clinical follow-up including patient validation of recovery further enhances risk communication for future vaccinees. The experience with post-smallpox vaccine myocarditis may inform future efforts to understand these reactions with other vaccines. Particularly the question of disability and the need for support is of critical importance to patients, families, and medical professionals caring for these patients with consideration of health insurance and loss of income. Finally, improved data quality is needed to inform the Countermeasure Injury Compensation Program and other programs to assist individuals with the long-term impact of an illness possibly related to vaccine-associated adverse events [58–60]. Combined, these efforts will improve understanding of the mechanisms and clinical implications of rare adverse events and contribute to precision medicine and novel approaches to risk mitigation.

There is a need for clinical expertise and resources to support the following: (1) enhanced surveillance, care, investigation, and data collection; (2) a national and international coordinated and collaborative platform for rapid capture and sharing of evolving vaccine safety/efficacy information; (3) the risk communication necessary to respond to the gamut of questions regarding vaccine safety and follow-up care. Assisting and educating providers in real time when rare, but serious, vaccine associated adverse events arise is a crucial service that is needed especially by healthcare workers in remote locations and those dealing with concerned loved ones as well as the patients experiencing potential complex vaccine-associated adverse events. An example of a new initiative embracing some of these challenges is the International Network of Special Immunization Services (INSIS). INSIS, as a part of the International Brighton Collaboration, has started developing collaboration between multinational special clinical immunization services for clinical case-based knowledge sharing and development of potential research networks to facilitate rapid visibility of clinical experience to improve both diagnosis and treatment of adverse events [61].

Improvements in national and international support for specialized clinical vaccine safety services coordinated with vaccine safety surveillance activities continue to be needed. Funding and training for special testing and evaluation of complex adverse events, including deaths, to support more precise causality assessments remain a significant shortfall.

## Supporting information

S1 Fig Bar chart of recovery time ranges shown by case type (myocarditis and pericarditis) with numbers. (PDF)

S1 Table MedDRA (Medical Dictionary for Regulatory Activities) TERMS for selection of cardiac VAERS. (PDF)

S2 Table Definitions for aggregate variables used in final demographic, clinical and laboratory data analyses. (PDF)

S3 Table Ejection fraction stratification and associated documentation of hypokinesis data. (PDF)

S4 Table Medication prescribed and cardiovascular disease risk factors for MP cases with comparison of patterns in myocarditis versus pericarditis. (PDF)

S5 Table Gender differences in clinical presentation and acute therapies prescribed. (PDF)

S6 Table Comparison of pericarditis cases by recovery rates (less than 1 year versus prolonged or no recovery after 1 year or longer) for baseline measures of cardiac injury and function. (PDF)

S7 Table Additional information about final adjudication of death case 3. (PDF)

S8 Table Drug and vaccine associated adverse event reports from 47 countries. (PDF)

## Abbreviations

AEFI: Adverse events following immunization
BMI: Body mass index
CAD: Coronary artery disease
CCATH: Cardiac catheterization
CDC: Centers for Disease Control and Prevention
CI: Confidence intervals
CK-MB: Creatine kinase-myoglobin binding
CMR: Cardiac magnetic resonance
CRP: C-reactive protein
DMSS: Defense Medical Surveillance System
DNA: Deoxyribonucleic acid
DOD: Department of Defense
ECG: Electrocardiogram
ECHO: Echocardiogram
EF: Ejection fraction
ESR: Erythrocyte sedimentation rate
FDA: Food and Drug Administration
IHD: Immunization Healthcare Division
IQR: Interquartile range
LV: left ventricular
MedDRA: Medical Dictionary for Regulatory Activities
MHS: Military Health System
MI: Myocardial infarction
MP: Myocarditis/Pericarditis
MRI: Magnetic resonance imaging
mRNA: Messenger RNA
NSAID: Non-steroidal anti-inflammatory
PCR: Polymerase chain reaction
RNA: Ribonucleic acid
SPV: Smallpox Vaccine
SSA/RO: Anti–Sjögren’s-syndrome-related antigen A autoantibodies
US: United States
VA: Veterans Administration
VAERS: Vaccine Adverse Events Reporting System
VHCN: Vaccine Healthcare Centers Network
WBC: White blood cell
WHO: World Health Organization
