# Supplementary material for: Myocarditis and pericarditis recovery following smallpox vaccine 2002–2016: A comparative observational cohort study in the military health system
Source: PLoS One. 2023 May 8;18(5):e0283988. doi: 10.1371/journal.pone.0283988 (PMC10166549; doi:10.1371/journal.pone.0283988)

**Figure 1s**: Bar chart of recovery time ranges shown by case type (myocarditis and pericarditis) with numbers

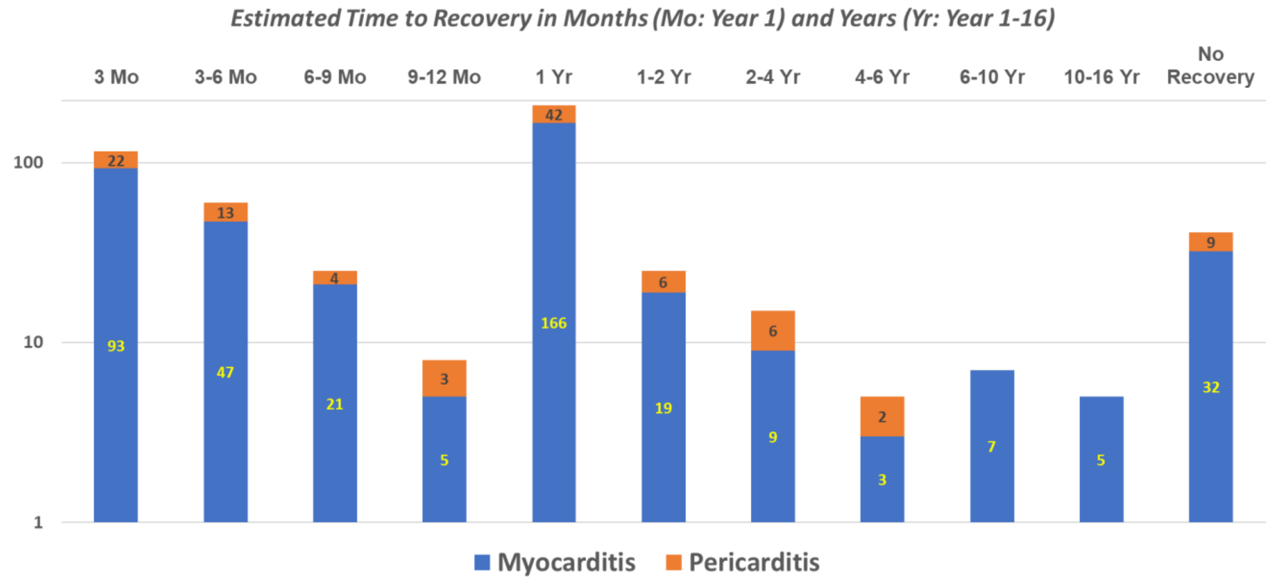

Supplement: S1 Fig — (PDF) [file pone.0283988.s001.pdf]
