# Supplementary material for: Myocarditis and pericarditis recovery following smallpox vaccine 2002–2016: A comparative observational cohort study in the military health system
Source: PLoS One. 2023 May 8;18(5):e0283988. doi: 10.1371/journal.pone.0283988 (PMC10166549; doi:10.1371/journal.pone.0283988)
Supplement: S1 Table — (PDF) [file pone.0283988.s002.pdf]

Table 1s: MedDRA (Medical Dictionary for Regulatory Activities) TERMS for Selection of Cardiac VAERS [1]

MedDRA Terms for Selection of Cardiac VAERS	
10002383 (ANGINA PECTORIS)	10002388 (ANGINA UNSTABLE)
10003119 (ARRHYTHMIA)	10003658 (ATRIAL FIBRILLATION)
10003662 (ATRIAL FLUTTER)	10007548 (CARDIAC ENZYMES INCREASED)
10008469 (CHEST DISCOMFORT)	10008479 (CHEST PAIN)
10013968 (DYSPNOEA)	10013969 (DYSPNOEA AT REST)
10013971 (DYSPNOEA EXERTIONAL)	10028596 (MYOCARDIAL INFARCTION)
10028600 (MYOCARDIAL ISCHAEMIA)	10028606 (MYOCARDITIS)
10028650 (MYOPERICARDITIS)	10033557 (PALPITATIONS)
10034484 (PERICARDITIS)	10058268 (TROPONIN I INCREASED)
10058267 (TROPONIN INCREASED)	10058269 (TROPONIN T INCREASED)
10047290 (VENTRICULAR FIBRILLATION)	10047294 (VENTRICULAR FLUTTER)
10047302 (VENTRICULAR TACHYCARDIA)	
