# Supplementary material for: Myocarditis and pericarditis recovery following smallpox vaccine 2002–2016: A comparative observational cohort study in the military health system
Source: PLoS One. 2023 May 8;18(5):e0283988. doi: 10.1371/journal.pone.0283988 (PMC10166549; doi:10.1371/journal.pone.0283988)
Supplement: S2 Table — (PDF) [file pone.0283988.s003.pdf]

Table 2s: Definitions for aggregate variables used in final demographic, clinical and laboratory data analyses

Data Variable Definitions	Description and Limitations
**Cardiac Symptoms**	Chest pain (including pain in neck, jaw, back or arm with or without radiation to forearm) and/or dyspnea (at rest and/or with exertion)
**Systemic Symptoms**	Headaches, body aches and/or fatigue (prodromal or concomitant systemic symptoms)
**Gastrointestinal Symptoms**	Nausea, vomiting, indigestion, heartburn, and/or abdominal pain.
**Race and ethnicity**	Data available often did not provide race separate from ethnicity and was heterogenous so this variable focused on white and black with others including those that only had Hispanic listed. This approach was also necessary for comparison to the background population immunized because that database did not have ethnicity and race data elements available as separate variables.
**Other vaccines given**	The number of other vaccine doses given within 30 days of the SPV date represented more than 30 different combinations and were summarized by a number of additional vaccines categorized by 0, 1 or >2.
**Non-steroidal anti-inflammatory**	Acute therapy included ibuprofen, naproxen, indomethacin, celecoxib, ketorolac, sulindac, etc. Data was not available for estimating specific duration of treatment in many cases. Excludes aspirin.
**Myocardial Infarction (MI) treatment**	Composite variable reflecting what acute treatment records indicated was the combination of therapies used with initial consideration of possible MI.
**Gastrointestinal (GI) treatment**	Composite of acute therapies addressing possible GI reflux as the etiology of the patient's symptoms.
**Dyslipidemia**	One or more of the following being abnormal as defined in the clinical note and/or in relation to the laboratory relevant population cut-off for normal levels: cholesterol, low-density lipoprotein (LDL), triglycerides and high-density lipoprotein (HDL). Elevated lipoprotein(a) was generally not available and so not included in this variable.
**Cardiac Risk Factors**	0 versus 1 or more excluding BMI<br>NOTE: SPV Program exemption from immunization if 3 or more cardiac risk factors were present
