# Supplementary material for: Myocarditis and pericarditis recovery following smallpox vaccine 2002–2016: A comparative observational cohort study in the military health system
Source: PLoS One. 2023 May 8;18(5):e0283988. doi: 10.1371/journal.pone.0283988 (PMC10166549; doi:10.1371/journal.pone.0283988)
Supplement: S3 Table — (PDF) [file pone.0283988.s004.pdf]

Table 3s: Ejection fraction stratification and associated documentation of hypokinesis data

Myocarditis/Pericarditis Case Review (%)	All Cases 348	Myocarditis 276	Pericarditis 72	P Value
**Cardiac Imaging Data**	**322**	**265**	**57**	
**Ejection fraction (EF) ≥60%**	148 (46.0)	116 (43.8)	32 (56.1)	0.09
**EF normal (>55%)**	222 (68.9)	174 (65.7)	48 (84.2)	
EF normal & hypokinesis	14/216 (6.5%)	13/171 (7.6%)	1/45 (2.2%)	0.31
**EF low normal (51-55%)**	53 (16.5)	47 (17.7)	6 (10.5)	
EF low normal & hypokinesis	9/49 (18.4%)	8/44 (18.2%)	1/5 (20.0%)	1.00
**EF ≤ 50% (Range: 19-50)**	47 (14.6)	44 (16.6)	3 (5.3)	**0.02**
EF ≤50% & hypokinesis	29/46 (63.0%)	27/43 (62.8%)	2/3 (66.7%)	1.00
**Hypokinesis**	52 (16.1)	48 (18.1)	4 (7.0)	**0.04**
**Abnormal: EF≤55% &/or Hypokinesis**	114 (35.4)	104 (39.2)	10 (17.5)	**0.002**
