# Supplementary material for: Myocarditis and pericarditis recovery following smallpox vaccine 2002–2016: A comparative observational cohort study in the military health system
Source: PLoS One. 2023 May 8;18(5):e0283988. doi: 10.1371/journal.pone.0283988 (PMC10166549; doi:10.1371/journal.pone.0283988)
Supplement: S4 Table — (PDF) [file pone.0283988.s005.pdf]

Table 4s: Medication prescribed and cardiovascular disease risk factors for MP cases with comparison of patterns in myocarditis versus pericarditis

**Myocarditis versus Pericarditis** (%)	**All Cases 348**	**Myocarditis 276**	**Pericarditis 72**	**P Value**
**Medications Prescribed**				
Data Available	**336**	**267**	**69**	
NSAID with Aspirin	289 (86.0)	232 (86.9)	57 (82.6)	0.36
Prescribed Aspirin	75 (22.3)	69 (25.8)	6 (8.7)	**0.002**
Prescribed NSAID (no aspirin)	212 (63.1)	161 (60.3)	51 (73.9)	0.04*
Prescribed Colchicine	55 (16.4)	46 (17.2)	9 (13.0)	0.40
Prescribed Other Meds	183 (54.5)	148 (55.4)	35 (50.7)	0.48
Prescribed Ketorolac	32 (10.0)	25 (9.4)	7 (10.1)	0.84
Prescribed Corticosteroids	8 (2.4)	7 (2.6)	1 (1.5)	1.00
Narcotics/Opioids Prescribed	55 (16.4)	49 (18.4)	6 (8.7)	0.05
Myocardial Infarction treatment	54 (16.1)	49 (18.4)	5 (7.3)	**0.03**
Gastrointestinal treatment	31 (9.2)	24 (9.0)	7 (10.1)	0.77
**Cardiac Risk Factors**	**348**	**276**	**72**	
Hypertension	24 (6.9)	20 (7.3)	4 (5.6)	0.80
Dyslipidemia	45 (12.9)	34 (12.3)	11 (15.3)	0.51
Family history (early CVD)	54 (15.5)	47 (17.0)	7 (9.7)	0.13
**Number of Risk Factors**				
• 2 or More Risk Factors	24 (6.9)	19 (6.9)	5 (6.9)	
• 1 Risk Factor	74 (21.3)	63 (22.8)	11 (15.3)	
• NO Cardiac Risk Factors	250 (71.8)	194 (70.3)	56 (77.8)	0.37

NSAID: non-steroidal anti-inflammatory drugs;
Tobacco recent or current use: 38.2% with no difference between myocarditis and pericarditis cohorts.
BMI $\geq$ 30%: 17.5% with no difference between myocarditis and pericarditis and with limitations in interpretation given unreliability of accuracy from calculated value (see Methodology).
Cardiovascular Disease (CVD) Risk Factors composite variable is defined as having hypertension and/or dyslipidemia, and/or diabetes mellitus, and/or family history of CVD [No one had diabetes at the time of vaccination].
Dyslipidemia: Included one or more of the following: elevated cholesterol >200 mg/dL; LDL>120 spell out NOT lipoprotein(a) elevation. objective definition describes hyperlipidemia as low-density lipoprotein (LDL), total cholesterol, triglyceride levels, or lipoprotein levels greater than the 90th percentile in comparison to the general population, or an HDL level less than the 10th percentile when compared to the general population. [2]
