# Supplementary material for: Myocarditis and pericarditis recovery following smallpox vaccine 2002–2016: A comparative observational cohort study in the military health system
Source: PLoS One. 2023 May 8;18(5):e0283988. doi: 10.1371/journal.pone.0283988 (PMC10166549; doi:10.1371/journal.pone.0283988)
Supplement: S5 Table — (PDF) [file pone.0283988.s006.pdf]

TABLE 5s: Gender differences in clinical presentation and acute therapies prescribed

**Myocarditis/Pericarditis Clinical Presentation** (%)	**All Cases**	**Female**	**Male**	**P Value**
**Total Cases**	**348**	**14**	**334**	
**Myocarditis**	276 (79.3)	4 (28.6)	272 (81.4)	
**Pericarditis**	72 (20.7)	10 (71.4)	62 (18.6)	**<0.0001***
**Time in Days Post Vaccine Date**				
Medical evaluation (IQR)<br>Range	11 (10,12)<br>1-48	10.5 (9,13)<br>6-35	11 (10,12)<br>1-48	0.85
**Cardiac Symptoms**				
Chest pain	339 (97.4)	14 (100.0)	325 (97.3)	1.00
Dyspnea	237 (68.1)	7 (50.0)	230 (68.9)	0.15
Palpitations	64 (18.4	4 (28.6)	60 (18.0)	0.30
Edema	2 (0.6)	0 (0.0)	2 (0.6)	1.00
Nausea	73 (21.0)	3 (21.4)	70 (21.0)	1.00
Positional/movement pain	224 (64.4)	11 (78.6)	213 (63.8)	0.39
**Cardiac Symptoms: Number**				
2 or more	264 (75.9)	10 (71.4)	254 (76.1)	
< 2	84 (24.1)	4 (28.6)	80 (24.0)	0.75
**Systemic/Other Symptoms**				
Fever/Chills	156 (44.8)	4 (28.6)	152 (45.5)	0.21
Diaphoresis	76 (21.8)	2 (14.3)	74 (22.2)	0.74
Headache	101 (29.0)	5 (35.7)	96 (28.7)	0.56
Myalgias/muscle aches	107 (30.7)	2 (14.3)	105 (31.4)	0.24
Fatigue	123 (35.3)	6 (42.9)	117 (35.0)	0.58
GI (nausea, vomiting, diarrhea, abdominal pain)	48 (13.8)	3 (21.4)	45 (13.5)	0.42
Upper respiratory symptoms	11 (3.2)	0 (0.0)	11 (3.3)	1.00
**Systemic symptoms:** other				
None	88 (25.3)	4 (28.6)	84 (25.1)	
1	74 (21.3)	3 (21.4)	71 (21.3)	
2 or more	186 (53.4)	7 (50.0)	179 (53.6)	0.94
**Medications Prescribed**	**336**	**13**	**323**	
NSAID (including aspirin)	289 (86.0)	10 (76.9)	279 (86.4)	0.40
Colchicine	55 (16.4)	1 (7.7)	54 (16.7)	0.70
Other Meds	183 (54.5)	5 (38.5)	178 (55.1)	0.24
Corticosteroids	8 (2.4)	0 (0.0)	8 (2.5)	1.00
Narcotics/Opioids	55 (16.4)	1 (7.7)	54 (16.7)	0.70
Myocardial infarction focus	54 (16.1)	2 (15.4)	52 (16.1)	1.00
Gastrointestinal treatment	31 (9.2)	1 (7.7)	30 (9.3)	1.00
