# Supplementary material for: Myocarditis and pericarditis recovery following smallpox vaccine 2002–2016: A comparative observational cohort study in the military health system
Source: PLoS One. 2023 May 8;18(5):e0283988. doi: 10.1371/journal.pone.0283988 (PMC10166549; doi:10.1371/journal.pone.0283988)
Supplement: S6 Table — (PDF) [file pone.0283988.s007.pdf]

Table 6s: Comparison of pericarditis cases by recovery rates (less than 1 year versus prolonged or no recovery after 1 year or longer) for baseline measures of cardiac injury and function

**Pericarditis Cohort (%)**	**All Cases**	**Recovery <1-year, Non-Impacting**	**Prolonged Impacting or No Recovery**	**P Value**
**Follow-up Data**	**306**	**200**	**106**	
Pericarditis	65 (21.2)	39 (19.5)	26 (24.5)	
**Time to Recovery**	**57**	**39**	**18**	
Median in years (IQR)<br>Range	0.41 (0.15,1.98)<br>0.11-7.91	0.19 (0.12,0.45)<br>0.11-0.98	3.48 (2.17,4.59)<br>1.42-7.91	
**Cardiac Injury**				
**Troponin Qualitative**	**52**	**29**	**23**	
Troponin Positive	12 (23.1)	5 (17.2)	7 (30.4)	0.26
**Troponin Quantitative**	**8**	**4**	**4**	
Troponin < 0.100	5 (62.5)	3 (75.0)	2 (50.0)	
Troponin ≥ 0.100 ng/ml	3 (37.5)	1 (25.0)	2 (50.0)	1.00
**Cardiac Imaging**				
**EF>50% versus ≤50%**	**52**	**29**	**23**	
EF > 50%	49 (94.2)	29 (100.0)	20 (87.0)	
EF ≤ 50%	3 (5.8)	0 (0.0)	3 (13.0)	0.08
**EF ≥60% & Changes**				
EF ≥60% after recovery	19/22 (86.4)	10/11 (90.9)	9/11 (81.8)	1.00
EF increased ≥8%	8/19 (42.1)	3/9 (33.3)	5/10 (50.0)	0.65
Hypokinesis	4 (7.7)	2 (6.9)	2 (8.7)	1.00
**Pericardial Imaging**				
Pericardial Effusion	5 (9.6)	4 (13.8)	1 (4.4)	0.37
**Cardiac Risk Factors**	**65**	**39**	**26**	
0	52 (80.0)	32 (82.0)	20 (76.9)	
1 or more	13 (20.0)	7 (18.0)	6 (23.1)	0.61
**Acute Medications**				
NSAID (and aspirin)	53/62 (85.5)	31 (86.1)	22 (84.6)	1.00
NSAID (not aspirin)	47/62 (75.8)	27/36 (75.0)	20/26 (76.9)	
Corticosteroid Therapy	1/63 (1.6)	1/37 (2.7)	0/26 (0.0)	1.00

EF: Ejection function; NSAID: non-steroidal anti-inflammatory drugs excluding aspirin.
