# Supplementary material for: Myocarditis and pericarditis recovery following smallpox vaccine 2002–2016: A comparative observational cohort study in the military health system
Source: PLoS One. 2023 May 8;18(5):e0283988. doi: 10.1371/journal.pone.0283988 (PMC10166549; doi:10.1371/journal.pone.0283988)
Supplement: S7 Table — (PDF) [file pone.0283988.s008.pdf]

Table 7s: Additional Information about final adjudication of death case 3

Relevant Data	Timeline	Specific Information
Prior Medical History	Day 0: Pre-SPV	18-year-old black male was in good health having completed basic training in Georgia during peak summer heat with extensive physical activity without difficulties (running 2 miles or more in greater than 100°F). He tolerated his initial immunizations (meningococcal PS, diphtheria-tetanus, inactivated polio vaccine hepatitis B) with no adverse effects. He had a fit-for-duty examination with documented normal blood pressure, BMI 24, no tobacco, alcohol or regular medication use. Glucose-6-phosphate dehydrogenase deficiency was documented.
Post-SPV	Day 8	Acute symptom onset during peak cytokine activation window for immune response to 1st dose of SPV [18]). Reported to family that he did not sleep well, was very thirsty all night and did not feel prepared for a longer run (5-miles). He denied feeling sick and decided to try the run.
Acute Events: Initial	Day 8 AM	Outdoor temperature was 45°F. During the initial run, he complained of bilateral leg cramps and that was the last thing he remembered during his interview after initial resuscitation.
Acute Events with Resuscitation	Day 8 late AM	He collapsed abruptly and came in and out of consciousness according to observers. Emergency services arrived: initial rhythm strip with narrow complex tachycardia (170-200 bpm). He was treated with intravenous adenosine 6 mg and then 12 mg due to no initial response. Emesis episode was followed by sedation with etomidate for onsite cardioversion 120 joules without response. The patient awoke briefly complaining of jaw and leg pains with dizziness and shortness of breath (respiratory rate 24/min). His initial Glasgow Coma Scale score was 12 (mild-moderate brain injury) with initial O2 saturation of 95-97%) He received 400 ml of normal saline and was transferred to the emergency room.
Emergency Room	Day 8 PM	Patient was combative and had a temperature of 102.9°F. He received an additional 2 liters of normal saline bolus, evaporating cooling measures and acetaminophen 950 mg. He had sinus tachycardia and an initial glucose of 52 mg/dL treated with an amp of D50 and associated improvement

		in mental status. Head CT and Chest x-ray normal. Stabilized and admitted for observation and care.
Clinical Course over 5 days		No evidence of specific infection (negative urine and blood cultures for bacterial, viral, fungal etiologies). Deteriorating clinical course with increasing liver enzymes, renal insufficiency and refractory cardiac arrest.
CASE REVIEW FINAL Consensus**		Pathology (2 major institutions) and Coroner: end-stage terminal heart no longer shows classic histologic evidence of myocarditis which is patchy in distribution and may be missed even early in the disease.
		Neuropathology: No evidence of encephalitis
		Immunohistochemical testing positive for orthopoxvirus at immunization site but NEGATIVE for other organ specimens (heart, lung, liver) and enlarged lymph node.
		Heart, liver and lung tissues PCR screening negative: enterovirus, human parvovirus B19, adenovirus, influenza A and B; liver negative for hepatitis B surface antigen and core antigen.
		Immunohistochemistry of the heart: moderate IgG deposition in the heart; quantitative autoantibody titers to cardiac myosin, beta-1,2 adrenergic receptors, M2 receptor mildly elevated.
		RYR1 genetic screen for hyperthermia risk: negative

****FOOTNOTES:**

1. **FINAL ASSESSMENT OF MEDICAL EXAMINER:** "Death is most likely due to myocarditis induced by immunologic consequences of the smallpox vaccination. The temporal relationship of the vaccination to the onset of illness, clinical history, laboratory results, and histology support this conclusion. It is likely that the 5-mile run precipitated the initial syncopal episode by increasing demand on the compromised heart. It is unlikely that hyperthermia played a role in this death, given that the temperature on the day of the run was less than 45 degrees, and that the decedent was young and healthy, and had completed basic training during the heat of the summer in Georgia. It is difficult to exclude a contribution from G6PD deficiency, but it is unlikely to have been significant, given that the decedent had been previously asymptomatic even under extreme physical stress. There is also no apparent role for G6PD deficiency in the development of myocarditis. It is possible that cross reactivity of anti-cardiac antibodies to skeletal muscle may have contributed to myoglobinuria, although there is no histologic correlation in skeletal muscle."
