# Supplementary material for: Myocarditis and pericarditis recovery following smallpox vaccine 2002–2016: A comparative observational cohort study in the military health system
Source: PLoS One. 2023 May 8;18(5):e0283988. doi: 10.1371/journal.pone.0283988 (PMC10166549; doi:10.1371/journal.pone.0283988)
Supplement: S8 Table — (PDF) [file pone.0283988.s009.pdf]

Table 8s: Drug and Vaccine Associated Adverse Event Reports from 47 Countries

**1967-2020: Individual Case Safety Reports from World Health Organization**	**Number of Reports**	**Percent %**	**Comments**
**Pharmacovigilance Database** (VigiBase)	21,185,309		
**Myocarditis: Suspected drug-induced cases**	6,823	0.0032	
Myocarditis, Suspected (62 drugs with 41 in 5 classes)	**5108**		
Antipsychotics	3108	60.1	
Salicylates	340	6.7	
Antineoplastic-cytotoxic	190	3.7	
Antineoplastic-immunotherapies	538	10.5	
Vaccines	790	15.5	
**Most Represented Drugs**	**5108**		
Clozapine	3035	59.4	
Immune checkpoint inhibitors	522	10.1	
Mesalazine (mesalamine or 5-aminosalicylic acid)	311	6.1	5-ASA
**Smallpox vaccine (live)**	**383**	**7.5**	Often with anthrax vaccine
**Myocarditis Vaccine Association**	790		**Reports with SPV**
Smallpox vaccine (SPV)	383	48.5	All
Influenza	181	22.9	26/181
Anthrax	163	20.6	147/163
Diphtheria, tetanus, pertussis with/without polio	148	18.7	
Hepatitis A &/or Hepatitis B	108	13.7	6/108
Typhoid	74	9.4	47/74
Meningococcal	54	6.9	
Tick born encephalitis	13	1.6	
Japanese encephalitis	17	2.2	6/17
Total Vaccines in Reports	1141		
SPV without other vaccines	200/383	52.2	

NOTE: Diphtheria, tetanus, pertussis, and/or polio vaccine (DTPP), Hepatitis A (HepA)and/or Hepatitis B (Hep B) vaccine.
